# *Salmonella* relies on siderophore exploitation at low pH

**DOI:** 10.1093/femsml/uqaf041

**Published:** 2026-01-14

**Authors:** Manon Ferry, Connor Sharp, Isabelle J Schalk, Olivier Cunrath

**Affiliations:** CNRS, UMR7242, Biotechnology and cell signaling, University of Strasbourg, 67412 Illkirch, France; School of Biological Sciences, Health and Life Sciences Building, University of Reading, RG6 6BX Reading, United Kingdom; CNRS, UMR7242, Biotechnology and cell signaling, University of Strasbourg, 67412 Illkirch, France; CNRS, UMR7242, Biotechnology and cell signaling, University of Strasbourg, 67412 Illkirch, France

**Keywords:** pH-dependent siderophore exploitation, *Salmonella*, siderophores, gut, iron, *fhuA*, *foxA*, *foxR*, *fur*, *rpoS*

## Abstract

*Salmonella enterica*, a prominent enteric pathogen, employs sophisticated iron acquisition mechanisms to overcome host-imposed iron limitation, notably through the production and uptake of siderophores—small, high-affinity iron-chelating compounds that scavenge iron from the host environment. In this study, we investigate how environmental pH influences *Salmonella’s* preference for its endogenous siderophores versus exogenous siderophores within the physiological range of the gastrointestinal tract. Through competition assays, gene expression analysis, and siderophore quantification, we demonstrate that *Salmonella* increasingly relies on exogenous siderophores under acidic conditions. This shift is attributed to reduced production of its endogenous siderophores, enterobactin and salmochelin. Deletion of the sigma factor RpoS enhances iron acquisition through increased endogenous siderophore production at low pH, suggesting a role in iron homeostasis regulation. Our findings reveal a pH-dependent difference in *Salmonella’s* iron acquisition strategy, highlighting the pathogen’s versatility in nutrient acquisition across varying gastrointestinal conditions. This research provides insights into *Salmonella’s* pathogenicity and may inform the development of targeted interventions for *Salmonella* infections.

## Introduction

Iron is a vital nutrient for all organisms, playing a key role as a cofactor in enzymes that drive growth and metabolism (Cairo et al. 2006). However, iron bioavailability is limited in many environments, making it a scarce resource for both the host and invading pathogens (Cook et al. 1986, Błażewicz et al. 2013, Palmer and Skaar 2016). During infection, the host organism mounts a defense strategy known as nutritional immunity, which tightly restricts the levels of available iron, effectively limiting its accessibility to pathogens (Yeh et al. 2004, Flo et al. 2004). By limiting access to iron, the host forces pathogens to compete for this essential resource. This competition shapes the course of infection and drives pathogens to develop various strategies for acquiring the iron they need to survive and proliferate. One of these strategies is the production of siderophores—high-affinity molecules that scavenge iron from the environment and facilitate its uptake into bacterial cells (Gao and Bian 2022). Siderophores are synthesized by various microorganisms in response to iron scarcity and are secreted into the extracellular environment, where they form stable complexes with iron (Schalk 2025). Once the siderophore-iron complex is formed, it is recognized and actively transported back into the microbial cell via specific transport systems. These transporters, located on the microbial surface, are highly selective and efficiently bring the iron-bound siderophore inside the cell, where the iron is released for cellular use (Schalk et al. 2012).

Numerous studies highlight the essential role of siderophores in the inflamed intestinal environment (Raffatellu et al. 2009, Deriu et al. 2013, Singh et al. 2015, Cunrath and Palmer 2021). The host actively interferes with bacterial siderophore utilization by producing molecules like lactoferrin, transferrin and calprotectin, which tightly bind iron, limiting its availability to pathogens (Aisen and Leibman 1972, Mazurier and Spik 1980, Nakashige et al. 2015). Additionally, the secretion of lipocalin-2 further restrict microbial access to iron, by capturing some iron-loaded siderophores (Goetz et al. 2002). Beyond the host’s defense mechanisms, the gut microbiota plays an additional role in the competition for iron. Certain commensal bacteria, such as *Lactobacillus* and *Bifidobacterium* species, along with some fungal strains like *Aspergillus* and *Malassezia*, are known to produce their own siderophores to acquire iron (Vazquez-Gutierrez et al. 2015, Vazquez-Gutierrez et al. 2017, Aguiar et al. 2021, Santus et al. 2022). Siderophores can also enter the intestinal environment through the diet, as they are produced by microorganisms naturally found in fermented foods (Ong and Neilands 1979, Santus et al. 2022). Together, these factors suggest that the intestinal environment is a dynamic landscape where iron is bound to a variety of siderophores. To thrive in this complex setting, many bacteria have evolved not only to produce their own siderophores but also to exploit exogenous siderophores—siderophores produced by other organisms and present in their environment (also referred to as xenosiderophores) (Luckey et al. 1972, Kramer et al. 2020, O’Brien et al. 2022). For instance, commensal gut bacteria such as *Bacteroides* can exploit siderophores produced by other microorganisms, as can gut pathogens like *Candida albicans, Clostridioides difficile* and *Escherichia coli* (Hartmann and Braun 1980, Heymann et al. 2002, Spiga et al. 2023, Hastie et al. 2023). Additionally, *Salmonella enterica* mutants that are unable to utilize exogenous siderophores have been shown to exhibit an attenuated phenotype following oral infection in chicks, cattle, or mice Chaudhuri et al. 2013). This dual capability to both produce and exploit siderophores provides pathogens with a versatile strategy to navigate the diverse ecological niches of the gut. To explore how these strategies may influence pathogen proliferation, we focus on *Salmonella enterica* subsp. *enterica* serovar Typhimurium, a prominent model pathogen of gastrointestinal infections and iron homeostasis (Cunrath and Palmer 2021).

*Salmonella* is capable of producing two catecholate-type siderophores—enterobactin (ENT), through the biosynthetic gene cluster EntCEBA (Pollack et al. 1970, Lundrigan and Kadner 1986), and salmochelin (SLC), a C-glycosylated derivative, through glucosylation mediated by the IroB enzyme (Fischbach et al. 2005)—in response to iron scarcity (Raymond et al. 2003). Of all reported siderophores so far, ENT exhibits the highest affinity for iron (K*_a_* = 10^49^ M^−1^) (Loomis and Raymond 1991) but can be neutralized by the host protein lipocalin-2, which sequesters ferric-ENT, thus blocking this iron acquisition pathway (Goetz et al. 2002). In contrast, the glucosylation of SLC (K*_a_*= 10^45^ M^−1^) (Raffatellu et al. 2009) prevents recognition by lipocalin-2, allowing *Salmonella* to evade this immune defense while maintaining its high affinity iron acquisition system, even under conditions of severe iron limitation (Fischbach et al. 2006).

**Figure 1.**
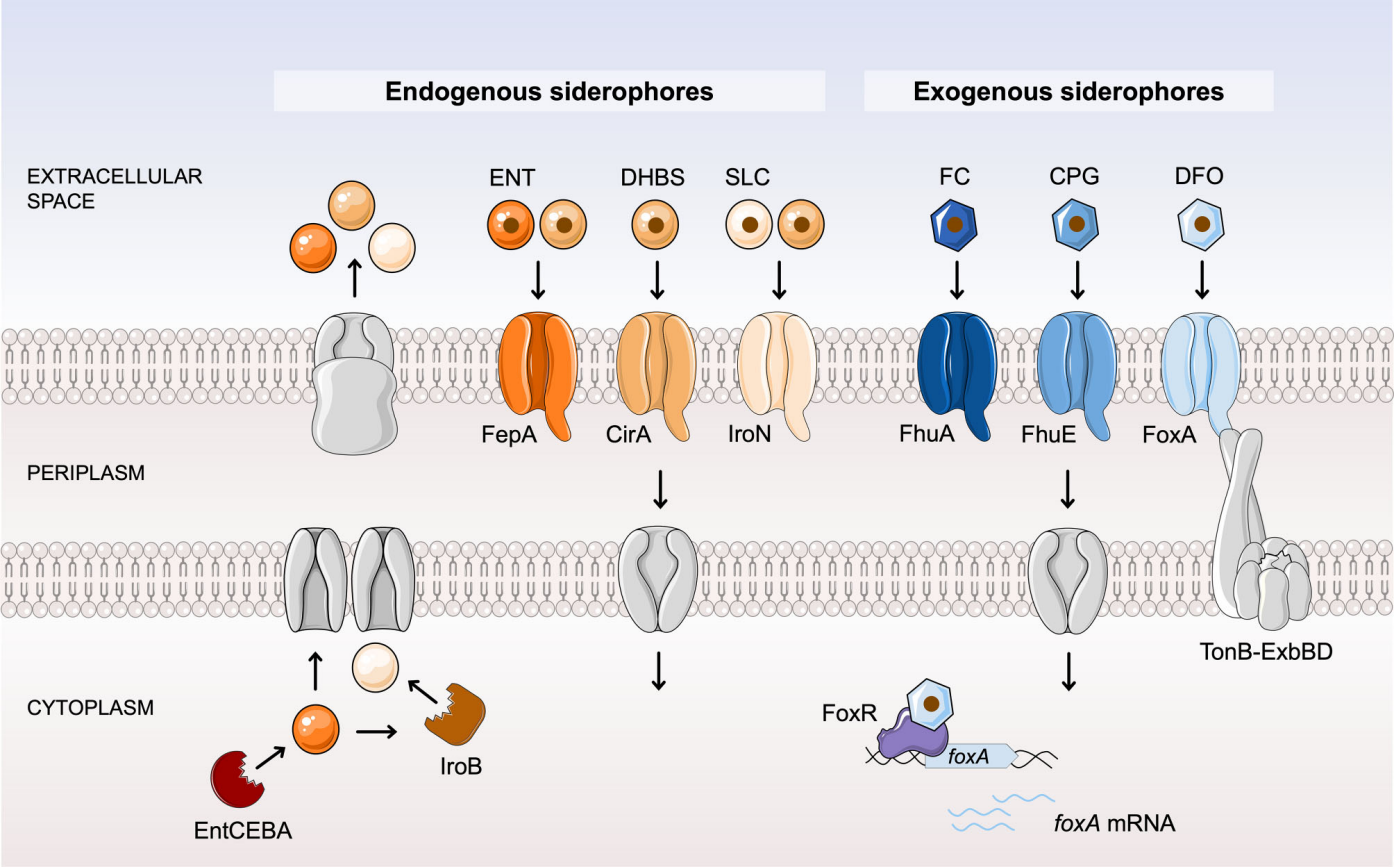
Siderophore mediated iron uptake in *Salmonella enterica*. Schematic diagram illustrating the pathways for the acquisition of endogenous (left) and exogenous (right) siderophores identified in *S. enterica* Typhimurium. Iron (brown circle) is captured from the extracellular environment and the ferri-siderophore complex is transported into the cell by TBDTs. Endogenous siderophores, produced by the *entCEBA* operon, include ENT (dark orange circle), its break-down product DHBS (medium orange circle), and SLC (light orange circle). SLC is a glycosylated derivative of ENT, with glycosylation facilitated by IroB. All three are transported by endogenous TBDTs: FepA for ENT and DHBS, CirA for DHBS and IroN for SLC and DHBS. Exogenous siderophores are FC (dark blue hexagon), CPG (medium blue hexagon), and DFO (light blue hexagon). They are imported by TBDTs FhuA, FhuE, and FoxA respectively, with FoxA being positively regulated by the cytoplasmic regulator FoxR.

As for all Gram negative bacteria, ferri-siderophore complexes formed in the bacterial environment cross the outer membrane through TonB-dependent transporters (TBDTs), utilizing energy derived from the proton motive force transmitted by the TonB-ExbB-ExbD inner membrane complex (Sauer et al. 1987, Rabsch et al. 1999, Postle and Larsen 2007, Wang et al. 2018, Kingsley et al. 1999). TBDTs are highly specific to their ligand, ensuring selective uptake of the iron-loaded siderophores (recently reviewed here (Cunrath and Palmer 2021, Schalk 2025)). In *Salmonella*, ferric-ENT, ferric-SLC, and their ferric break-down products 2,3-dihydroxybenzoylserine (DHBS) are recognized by specific TBDTs: FepA for ENT and DHBS, IroN for SLC and DHBS, and CirA for DHBS (Fig. 1, left part) (Lundrigan and Kadner 1986, Hantke 1990, Bäumler et al. 1998). Beyond producing its own endogenous siderophores, *Salmonella* can also exploit three exogenous siderophores found in its environment: ferrichrome (FC) (K*_a_*= 10^29^ M^−1^) (Schwarzenbach and Schwarzenbach 1963, Luckey et al. 1972), coprogen (CPG) (K*_a_*= 10^27^ M^−1^) (Wong et al. 1983, Sauer et al. 1987), and ferrioxamines (DFO) (K*_a_*= 10^32^ M^−1^) (Anderegg et al. 1963, Kingsley et al. 1999), all of which are hydroxamate-type siderophores (Fig. 1, right part). This specialized transport mechanism enables *Salmonella* to efficiently acquire iron from the environment, particularly under iron-limited conditions, by facilitating the uptake of the strong affinity ferric-siderophore complexes.

In *Salmonella*, iron homeostasis and more specific genes encoding siderophore transporters or involved in their biosynthesis are primarily regulated by a coordinated network involving the ferric uptake regulator (Fur), the small RNAs RyhB1 and RyhB2 and the stress response sigma factor RpoS. Fur acts as a key transcriptional repressor of iron acquisition genes under iron-replete conditions (Bagg and Neilands 1987), by binding to specific DNA sequences known as Fur boxes located in the promoter regions of target genes when complexed with Fe²⁺. During iron limitation, RyhB1 and RyhB2 are expressed and help conserve iron by downregulating nonessential iron-using proteins, while also stabilizing and activating the translation of certain iron uptake genes, such as *iroN* (Balbontín et al. 2016). RpoS, a central regulator of the general stress response, facilitates the expression of stress-related genes and has been shown to relieve Fur-mediated repression of genes involved in iron and manganese metabolism, thereby contributing to the dynamic regulation of the *Salmonella* iron content under stress conditions (Hall and Foster 1996). In addition, certain genes involved in siderophore acquisition are controlled by more specific mechanisms. For example, the TBDT FoxA, responsible for ferrioxamine E (DFOE) uptake, exhibits a ligand-specific upregulation mechanism mediated by the AraC-type transcriptional regulator FoxR. FoxR binds to DFOE in the cytoplasm, activating the transcription of the *foxA* gene and thereby upregulating FoxA expression to improve *Salmonella*’s access to this exogenous siderophore (Fig. 1, right part), thus optimizing the bacterial iron acquisition process under iron-limited conditions (Saldaña-Ahuactzi and Knodler 2022).

Our understanding of how pathogens selectively use siderophores under various infection conditions remains limited. One key question is whether pathogens primarily rely on the production of their own siderophores or whether they exploit exogenous ones, and how this choice is influenced by the dynamic conditions of the intestinal environment. Environmental factors such as changes in pH, which fluctuate within the gut, could significantly impact iron availability and may affect the way siderophores are utilized (McNeil et al. 1987, Valdebenito et al. 2006, Maurer et al. 2015, Wang et al. 2018). Given the energetic cost of siderophore production and the nutrient restrictive environment imposed by both the host and the innate microbiome, it is plausible to assume that, under certain conditions, pathogens might favor the exploitation of exogenous siderophores (Li et al. 2023). This raises important questions about the adaptive mechanisms’ pathogens employ to optimize their iron acquisition in the face of a constantly shifting gut environment. Here we show that siderophore exploitation is a vastly conserved strategy among all tested *Salmonella* sub-populations. We further show that exogenous siderophore exploitation is key for *Salmonella’s* survival during iron starvation at low pH. Despite complex regulatory mechanisms involved in iron homeostasis, we find that this pattern can be explained by a simple underlying principle: a stark reduction of the endogenous siderophore production in acidic conditions. Furthermore, we have shown that this down-regulation may represent a pathogen’s weakness that can be utilized for better pathogen control strategies (Santus et al. 2022, O’Brien et al. 2023, Shao et al. 2023, Gräff and Barry 2024).

## Results

### Conservation of siderophore-related genes across the *Salmonella enterica* species highlights their ecological importance

**Figure 2.**
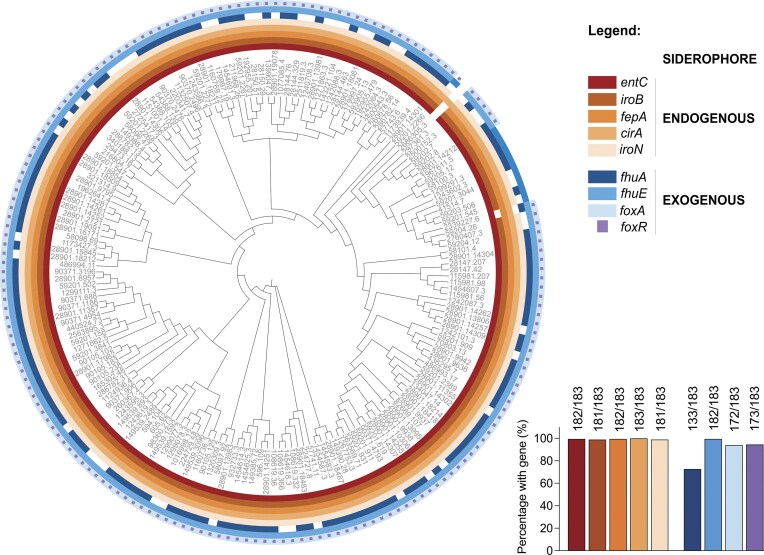
Conservation of siderophore-related genes across *Salmonella enterica* species highlights their ecological importance. Cladogram of 183 *Salmonella* strains, based on the concatenated alignment of universal core genes shared across all strains. Strains were selected to maximize genetic diversity with each strain having a unique MLST profile. Coloured rings depict the presence or absence of different siderophore related genes. The bar plot shows the percentage of strains that possess each studied gene.

*Salmonella enterica* is well known for its ability to both produce its own siderophores and exploit exogenous siderophores. *Salmonella* synthesizes ENT (via EntCEBA (Pollack et al. 1970, Lundrigan and Kadner 1986)) and SLC (via IroB (Fischbach et al. 2005)) which are recognized by specific TBDTs: FepA, IroN and CirA (Lundrigan and Kadner 1986, Hantke 1990, Bäumler et al. 1998) (Fig. 1). To assess the conservation of *Salmonella’s* endogenous siderophore uptake systems, we analyzed siderophore-related genes in 183 *S. enterica* strains. These strains were randomly chosen with one strain selected for each unique multilocus sequence typing (MLST) profile on the BV-BRC database to ensure a diverse and representative dataset (Wattam et al. 2017). Our results reveal a strong conservation of all endogenous siderophore biosynthesis and uptake systems across the species (Fig. 2). Nearly all strains possess the *entC* and *fepA* genes (>99%), which enable the biosynthesis and uptake of ENT, respectively, highlighting the importance of these genes in *Salmonella*’s iron acquisition strategies. Likewise, *cirA* is universally present, supporting the consistent use of this transporter across strains. Furthermore, the high conservation of both *iroB* and *iroN* (98.9%) in almost all strains suggests a strong conservation of the capacity to produce and import SLC.

In addition to its own siderophores, *Salmonella* can exploit the three exogenous siderophores FC, CPG, and DFO which are transported by FhuA, FhuE and FoxA, respectively (Sauer et al. 1987, Wang et al. 2018, Kingsley et al. 1999). Additionally, FoxR enhances DFOE uptake via FoxA induction (Saldaña-Ahuactzi and Knodler 2022). When analyzing the conservation of genes encoding for exogenous siderophore TBDTs as described above, we could show that although their conservation was slightly more variable than for the endogenous transporters, conservation still remains high (Fig. 2). The *fhuA* gene, responsible for FC transport, is slightly less conserved but is still present in about 73% of the strains. Conversely, the genes for CPG and DFO transport—*fhuE* (> 99%) and *foxA* (94%), respectively—are highly conserved, as is *foxR* (95%), the positive regulator of *foxA*. All but one strain possesses at least one exogenous siderophore transporter, underscoring the species-wide dependency to access exogenous siderophores in diverse environments (Fig. 2). A broader analysis of approximately 1 200 *Salmonella enterica* strains with unique genomes showed the same trend, reinforcing the conclusion that siderophore-dependent iron uptake systems are highly conserved across the species and likely play a key role in *Salmonella*’s infection process (Fig. S1; Table S5).

### The reliance of *Salmonella* on exogenous siderophore exploitation increases in acidic conditions

Changes in pH within the gastrointestinal tract can significantly affect siderophore physicochemical properties (Albrecht-Gary and Crumbliss 1998, Valdebenito et al. 2006). As the pH fluctuates, the chelating properties of siderophores may be modified, which in turn may influence their relative importance in iron acquisition. For example, ENT, a catecholate-type siderophore, sees its affinity strongly decrease upon acidification of the environment, due to protonation of the catechol groups (Loomis and Raymond 1991, Valdebenito et al. 2006). On the other hand, hydroxamate siderophores like DFO depict a weaker affinity for iron at neutral pH but show a weaker decrease in affinity upon acidification (Ihnat et al. 2002, Valdebenito et al. 2006). Given this pH-dependent variability in siderophore affinity, we hypothesize that acidic conditions in the gut could influence *Salmonella*’s preference towards exploiting exogenous hydroxamate siderophores, as their uptake might be less affected by the changes in iron affinity compared to their endogenous siderophore ENT.

**Figure 3.**
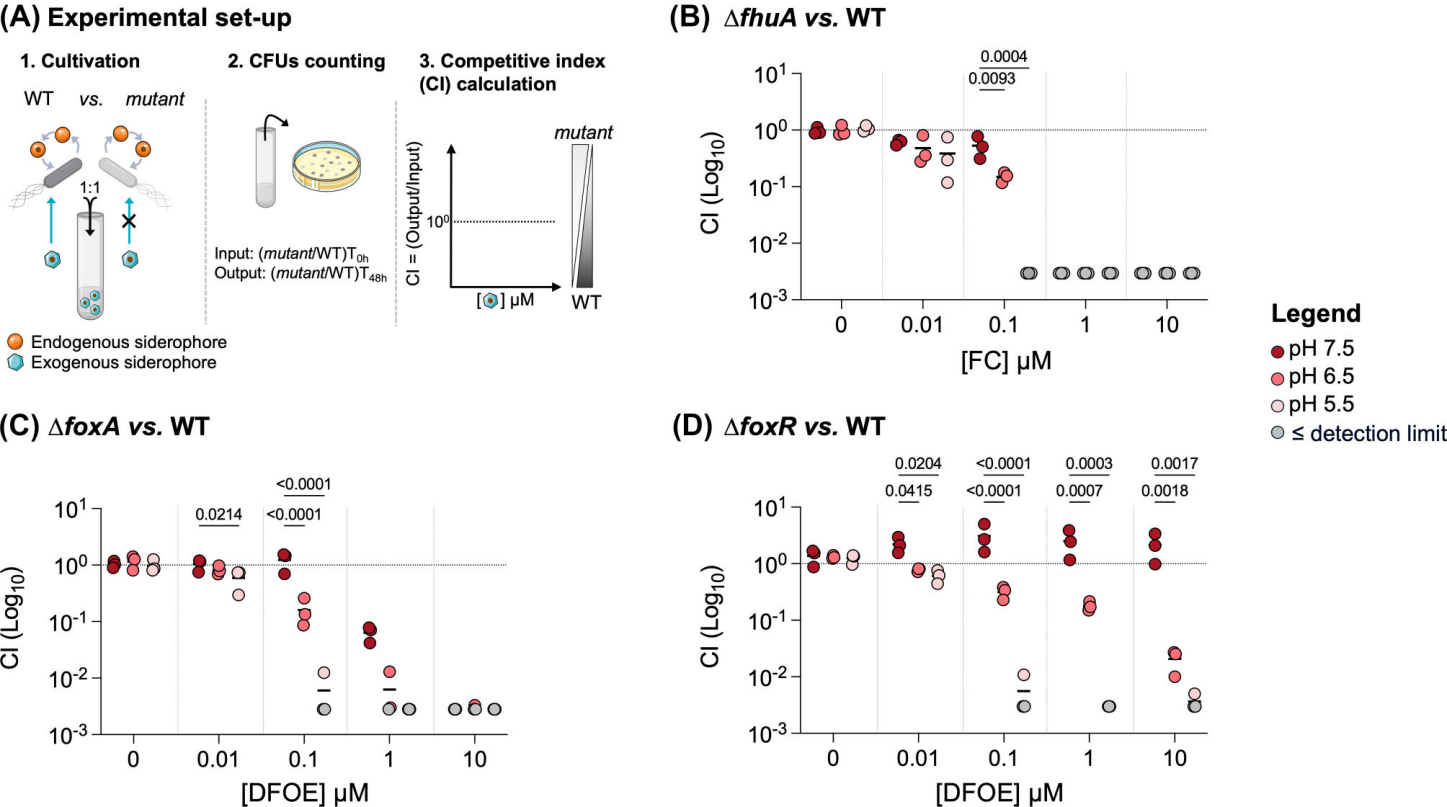
The reliance of *Salmonella* on exogenous siderophore exploitation increases in acidic conditions. **A**. Experimental setup for competition assays. Two fluorescently labeled *Salmonella* strains are mixed in equal amounts, with one strain unable to exploit the exogenous siderophore. Cultures are grown in Minimal Medium (MM) at pH 7.5 (dark red), 6.5 (medium red), or 5.5 (light red), with or without increasing concentrations of exogenous siderophore. After 48 hours, the ratio of mutant to WT colonies is determined by plating on selective agar, and the competitiveness index (CI) is calculated (for more details the Method section). A CI > 1 indicates a competitive advantage for the mutant strain, while a CI < 1 indicates an advantage for the WT strain. **B**. Competitive indexes of Δ*fhuA vs*. WT strain in presence of FC. **C**. Competitive indexes Δ*foxA vs*. WT strain in presence of DFOE. **D**. Competitive indexes of Δ*foxR vs*. WT strain in presence of DFOE. Each data point represents one biological replicate (n = 3). Points below the detection limit for each assay are shown in gray. Statistical analysis was performed using a two-way ANOVA followed by Sidak’s multiple comparisons test. Exact *P*-values are reported for all comparisons with *P* < 0.05 considered statistically significant.

To investigate the influence of pH on *Salmonella*’s use of exogenous siderophores, we performed bacterial competition assays (Fig. 3A). In these assays we investigated two different exogenous siderophores uptake systems; the FC uptake, in which the transcription and consequently the expression of the TBDT FhuA is solely regulated by the general iron regulator Fur; and the DFO uptake, where the transporter FoxA has its expression regulated by Fur and further induced by cytoplasmic DFOE via FoxR (Fig. 1) (Saldaña-Ahuactzi and Knodler 2022, Kingsley et al. 1999). A wild-type (WT) strain and an isogenic strain lacking the gene encoding for the exogenous-siderophore transporter (Δ*fhuA* or Δ*foxA*) or the regulator (Δ*foxR*) were co-cultured in iron-depleted media at various pH levels, with increasing concentrations of the corresponding exogenous siderophores. It is important to note that both competing strains were capable of producing and using their endogenous siderophores to acquire iron. For each condition, we measured the competitive index (CI) of the mutant strain relative to the WT strain (as calculated by the CFU ratio (mutant/WT) at 48 h divided by the CFU ratio (mutant/WT) of the inoculum) to assess the advantage conferred by the ability to exploit exogenous siderophores across different pH levels (Fig. 3A). These experiments showed a decreased fitness of the mutant strain in increasing concentrations of our exogenously added siderophores (Fig. 3BCD). At intermediate concentration such as 0.1 μM, this decrease was significantly more pronounced at low pH. Therefore, our results showed a significant pH-dependent effect on the advantage provided by FC exploitation (Fig. 3B). The inability to use FC, due to the deletion of *fhuA*, demonstrated that *Salmonella* gains a distinct advantage from FC exploitation at lower FC concentrations in acidic conditions than at neutral pH. Similarly, when comparing the WT strain that can use DFOE to the Δ*foxA* mutant strain that cannot, we observed the same pH-dependent advantage (Fig. 3C). Since FoxA appears more important under acidic conditions, we investigated the relative advantage of FoxR-mediated upregulation of the FoxA transporter. Our findings indicate that, in the presence of all tested concentrations of DFOE, FoxR-mediated upregulation is only relevant for *Salmonella* in acidic conditions (Fig. 3D). Growth assays revealed a striking pH-dependent role for FoxR. At neutral pH, a Δ*foxR* mutant grows similarly to WT, even in the presence of 10 µM of DFOE, indicating that FoxR is dispensable under these conditions (Fig. S2). In contrast, at acidic pH, the same Δ*foxR* mutant exhibits a significant growth defect compared to WT when DFOE is present, demonstrating that FoxR is essential for *Salmonella’s* fitness in acidic environments (Fig. S2). The same findings also hold in an Δ*entC* mutant background, unable to produce its own siderophore (Fig. S3). These findings reveal that acidic pH represents a condition in which the regulator FoxR becomes crucial for iron acquisition. At low pH, *Salmonella* requires both the FoxA transporter and its up-regulation by FoxR to effectively exploit DFOE siderophore; without which siderophore exploitation may be blocked.

### Reduced endogenous siderophore production increases dependence on exogenous siderophores in acidic conditions

While the affinity between iron and the siderophore varies at different pH, we explored if our pH-dependent phenotype could be explained by more than just the affinity difference between iron and the siderophores (Valdebenito et al. 2006). TBDTs that import siderophores are highly specific, with each transporter recognizing a particular iron-siderophore complex (Schalk 2025). Previous studies have shown that pH can alter the affinity of the siderophore-iron complex for its transporter (Li et al. 2023). To understand why *Salmonella* gains a significant growth advantage from exogenous siderophore utilization in acidic conditions, we examined whether pH affects the affinity of the siderophore-iron complex for its transporter. Therefore, we measured the affinity of Fe-DFOE, one of the three exogenous siderophores utilized by *Salmonella*, for its transporter FoxA. Specifically, *Salmonella* cells expressing FoxA were incubated with increasing concentrations of radiolabeled ^55^Fe-DFOE at 4°C in the presence of the proton motive force inhibitor CCCP. In such a condition, Fe-siderophores bind to their TBDT but no uptake into the bacteria occurs (Hoegy et al. 2005). After incubation, unbound complexes were removed by filtration and the radioactivity associated with the bacterial pellet was measured by scintillation counting. These data were used to generate binding curve and calculate the dissociation constant (*K_d_*), providing a quantitative measure of the interaction between DFOE-Fe and FoxA. Our findings indicate that the growth advantage is not due to changes in siderophore binding properties, as DFOE displayed consistent affinity for its transporter FoxA at both pH 7.5 and 5.5 with *K_d_* of around 3.2 ± 7.6 nM and 3.2 ± 11.0 nM, respectively (Fig. S4).

We then explored two other possible scenarios to explain the increased reliance on exogenous siderophores: either exogenous uptake systems are more highly expressed in acidic environments, or endogenous uptake systems are less expressed, leading to greater dependence on exogenous siderophore exploitation. To assess the expression levels of siderophore-uptake pathways in relation to pH, we used plasmid-encoded fluorescent transcriptional reporter fusions (Cunrath and Bumann 2019) in which we have cloned the promoter region of the gene of interest in front of GFP.

**Figure 4.**
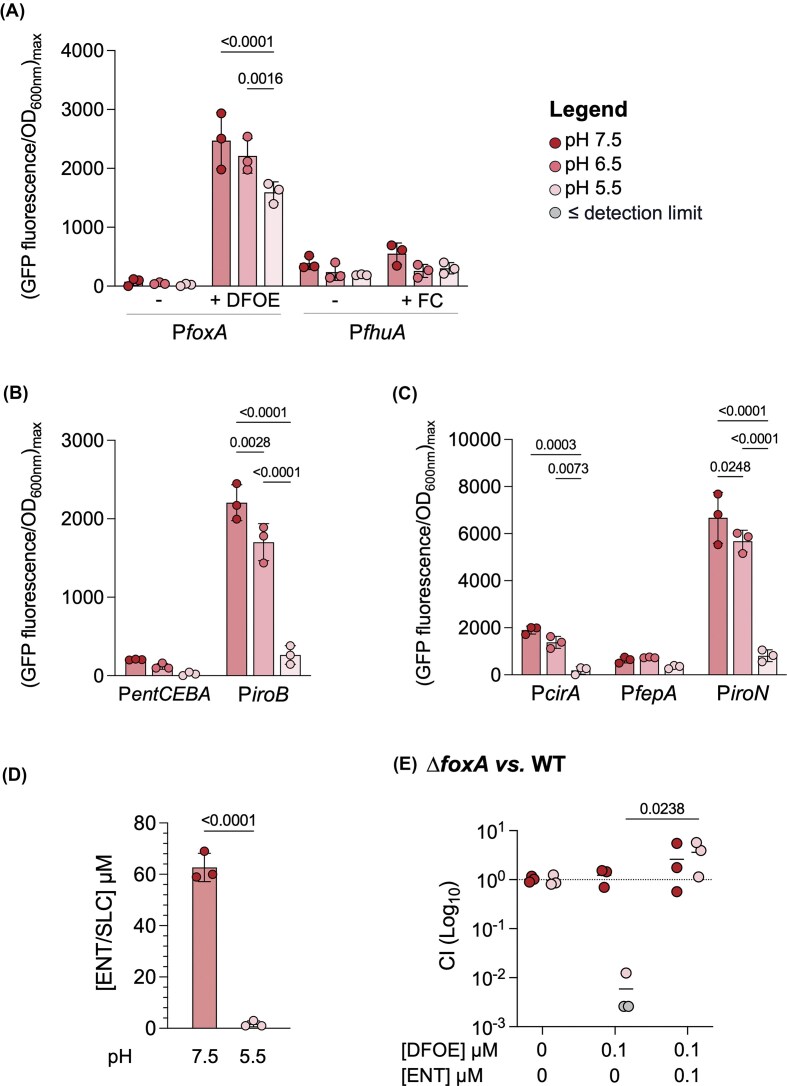
Endogenous siderophore production decreases in acidic conditions, heightening reliance on exogenous siderophores. **A–C**. Transcriptional expression of siderophore-related genes. WT strains carrying plasmid encoded fluorescent reporters were cultivated in MM at pH 7.5 (dark red), 6.5 (medium red), or 5.5 (light red). GFP-fluorescence emission data, normalized to optical density at 600 nm (OD_600nm_) of the culture, are shown as the maximum fluorescence per OD_600nm_. Each point represents a biological replicate. **A**. Expression levels of genes encoding exogenous siderophore transporters with or without 10 µM of their respective ligand. **B**. Expression levels of genes encoding enzymes involved in the biosynthesis and **C**. transporters involved in the uptake of endogenous siderophores. **D**. Concentration of endogenous siderophores (ENT/SLC, and DHBS) in the supernatant of WT *Salmonella* cultures after 24 hours of cultivation in MM at pH 7.5 (dark red) and 5.5 (light red). The concentrations of ENT and SLC are shown together, as they have similar spectral properties, with SLC being a glycosylated derivative of ENT. **E**. Competitive indexes of Δ*foxA vs*. WT strain at pH 7.5 5 (dark red) and 5.5 (light red). The competition was conducted in MM with no supplementation, 0.1 μM DFOE or 0.1 μM DFOE plus 0.1 μM ENT. The output ratio was calculated after 48 hours of culture. Each data point represents one biological replicate (n = 3). Points below the detection limit for each assay are shown in gray. Statistical analysis was performed using a one-way ANOVA followed by Tukey’s multiple comparisons test for panels A–C, an unpaired two-tailed t-test for panel D and two-way ANOVA with Sidak’s multiple comparisons test for panel E. Exact *P*-values are reported for all comparisons with *P* < 0.05 considered statistically significant.

Using this approach, while no difference was observed in our control promoter (Fig. S5A), we observed a reduction in the transcription of the exogenous *foxA* and *fhuA* genes under acidic conditions, even when grown in the presence of their respective ligand (Fig. 4A). We then investigated the expression of genes involved in the endogenous siderophore uptake system (Fig. 4B-C). Specifically, the expression of the *entCEBA* operon, which is responsible for ENT production, and *iroB*, which contributes to SLC production, were also reduced in acidic conditions, confirming previous findings in which a reduced *iroA* expression was observed at pH 5.5 (Foster et al. 1994). Similarly, the expression of endogenous siderophore TBDTs such as *cirA, fepA*, and *iroN* decreased, suggesting that endogenous siderophores are produced at lower levels in acidic conditions. This was further supported by the quantification of endogenous siderophores using UV-visible spectroscopy, which revealed reduced concentrations of endogenous siderophores (ENT/SLC and DHBS) in the supernatant of strains cultivated under acidic conditions (Fig. 4D). To make sure that this phenomenon is not strain-specific, we quantified ENT/SLC and DHBS production in various phylogenetically diverse *Salmonella* strains, which reveal similar trends (Fig. S6). In addition to the use of siderophores, *Salmonella* also possesses ferrous iron uptake systems such as FeoABC, SitABCD and MntH (Makui et al. 2000, Kehres et al. 2002, Hantke 2003) (recently reviewed here (Cunrath and Palmer 2021)). We examined whether the expression of these three major ferrous iron import systems is upregulated under acidic conditions, however, our results indicate that these import systems are not significantly more expressed in acidic environments compared to neutral environments (Fig. S7). We observe a lower expression profile of *sitABCD* at lower pH, confirming previous findings (Kehres et al. 2002). While our data suggest no difference in ferrous iron uptake system expression at low pH, this does not rule out increased uptake, since Fe²⁺ is more soluble and thus more available in acidic conditions. Taken together, these findings suggest that, under acidic conditions, *Salmonella* has a greater reliance on exogenous siderophores due to reduced production of its own endogenous siderophores and a change in iron-chelating affinity, despite also reducing exogenous transporter expression. At neutral pH, ENT/SLC siderophores have a higher affinity for iron than hydroxamate siderophores, but at acidic pH, their affinity decreases to a level comparable to that of hydroxamates, making them less competitive in acidic conditions (Valdebenito et al. 2006). In our competition model, hydroxamate siderophores are already present in the environment, which may further enhance *Salmonella*’s dependence on these exogenous siderophores to access iron. This combination of limited production and reduced affinity in acidic conditions likely drives *Salmonella* to favor exogenous siderophore uptake. To test whether this increased reliance is indeed due to reduced endogenous siderophore production, we supplemented the culture medium with pure ENT, the endogenous siderophore of *Salmonella*, to see if this addition could alleviate the dependence on exogenous sources (Fig. 4E). We competed a WT strain with a strain deficient in DFOE uptake (Δ*foxA* mutant) at both pH 7.5 and 5.5. While the addition of DFOE alone conferred a growth advantage to the WT strain in acidic conditions, this advantage was lost when the same concentration of ENT was added alongside DFOE. The presence of exogenous ENT effectively reduces *Salmonella*’s reliance on exogenous siderophores, highlighting the critical role of endogenous siderophore production in facilitating growth under acidic conditions.

Our results indicate that *Salmonella*’s increased reliance on exogenous hydroxamate-type siderophore exploitation under acidic conditions is driven by a combination of factors. The decreased production of endogenous catechol siderophores, combined with the reduced affinity of *Salmonella*’s endogenous siderophores for iron in acidic environments, drives a greater dependence on exogenous sources of siderophores.

### Loss of the sigma factor RpoS enhances endogenous siderophore production and iron acquisition at acidic pH

To elucidate the regulatory mechanisms underlying the reduced production of endogenous siderophores in acidic conditions, we investigated the roles of two potential candidates: Fur and RpoS (σ^S^). Fur is a key regulator of iron homeostasis in *Salmonella*, typically repressing iron import systems when iron is plentiful (Bagg and Neilands 1987). RpoS is a sigma factor involved in the transcription of genes associated with stress responses, such as acid stress (Lange and Hengge-Aronis 1991, Soo Lee et al. 1995). Given that our experimental conditions are both iron-poor and acidic, both Fur and RpoS were considered promising candidates for this study. Notably, the stress sigma factor RpoS has been shown to counteract Fur’s repression of genes involved in iron and manganese metabolism and modulate the metallome of *Salmonella enterica* serovar Typhimurium (Hall and Foster 1996). Therefore, a potential regulatory interplay between Fur and RpoS in response to environmental stressors such as acidic conditions has been suggested, where both factors could play a role in modulating iron homeostasis and siderophore production, hence our rational to examine the implication of both key regulators in the modulation of our phenotype.

**Figure 5.**
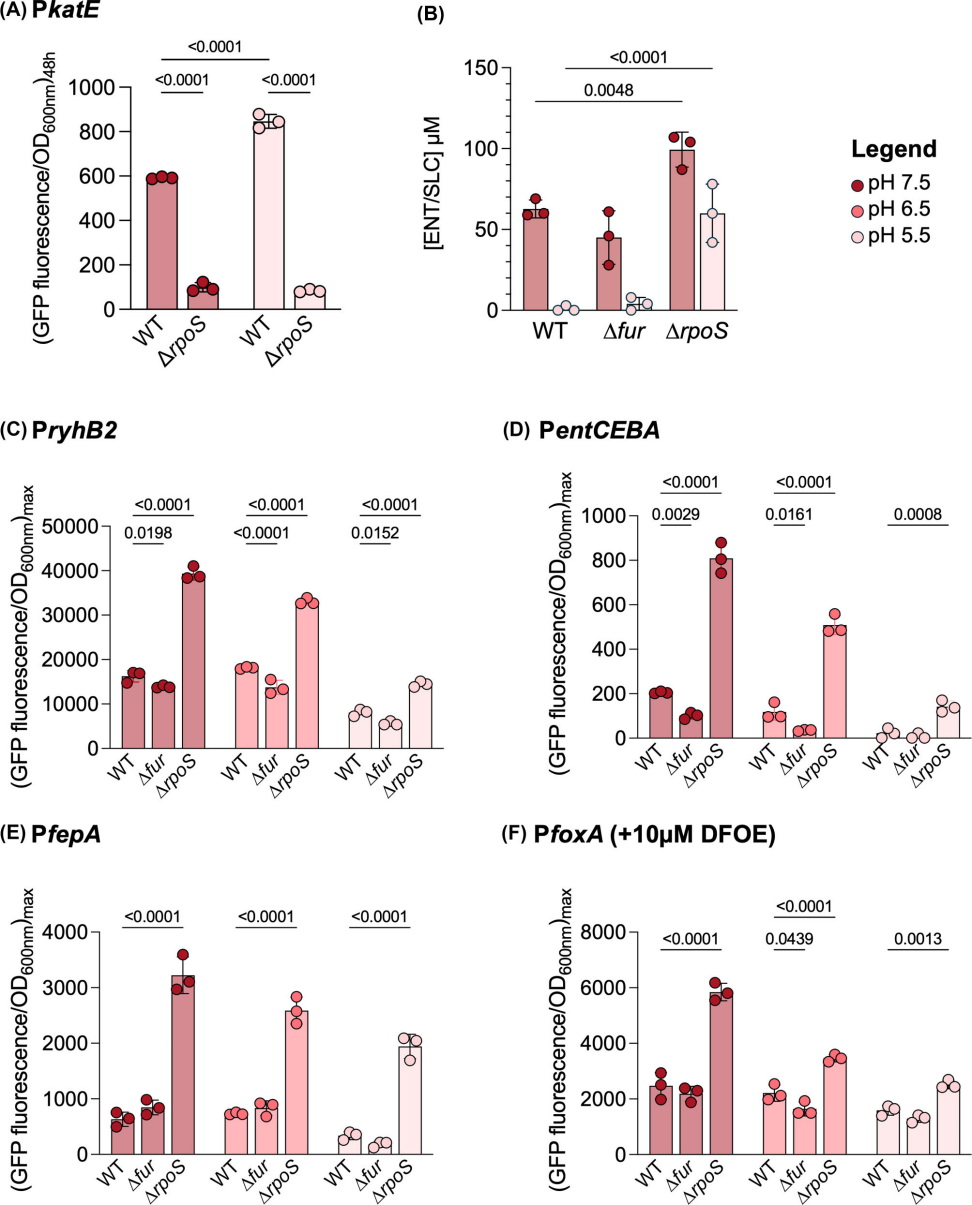
Loss of the sigma factor RpoS enhances endogenous siderophore production and acquisition at acidic pH. **A**. Measurement of RpoS activity using P*katE* in a WT and ∆*rpoS* mutant strain grown in LB at pH 7.5 (dark red) and 5.5 (light red). **B**. Measurement of endogenous siderophore production (ENT/SLC) in the ∆*fur* and ∆*rpoS* mutants grown in MM at pH 7.5 (dark red) and 5.5 (light red). Siderophore concentrations were quantified from the supernatant after 24 hours of cultivation (n = 3). **C–F**. Transcriptional expression of siderophore-related genes in ∆*fur* and ∆*rpoS* mutants. Strains were cultivated in MM at pH 7.5 (dark red), 6.5 (medium red), or 5.5 (light red). GFP-fluorescence emission data, normalized to optical density at 600 nm (OD_600nm_) of the culture are shown as the maximum fluorescence per OD_600nm_. Each point represents one biological replicate (n = 3). **C**. Expression of the iron deprivation marker *ryhB2* (P*ryhB2*). **D**. Expression of the operon involved in endogenous siderophore biosynthesis *entCEBA* (P*entCEBA*). **E**. Expression of the transporter of ENT *fepA* (P*fepA*). **F**. Expression of the transporter of ferrioxamine E *foxA* (P*foxA*). Statistical analysis was performed using two-way ANOVA with Sidak’s multiple comparisons test for panels A–B and one-way ANOVA followed by Tukey’s multiple comparisons test for panels C-F. Exact *P*-values are reported for all comparisons with *P* < 0.05 considered statistically significant.

Firstly, we measured RpoS activity using a commonly used *rpoS*-reporter gene (P*katE*) (Tanaka et al. 1997) at pH 7.5 and 5.5, which expression is driven by RpoS. We could show that RpoS activity was slightly higher at lower pH and completely abolished in a Δ*rpoS* mutant (Fig. 5A), suggesting a slight increase in RpoS activity at pH 5.5. Additionally, we could show that intracellular iron concentrations were not significantly different at pH 7.5 and 5.5 (Fig. S5B). In order to investigate whether Fur and/or RpoS were key regulators at the origin for the decreased ENT production at pH 5.5, we generated isogenic mutant strains for both regulators. We then measured endogenous siderophore concentrations in the supernatants of strains grown at pH 7.5 and 5.5 for both Δ*fur* and Δ*rpoS* mutant. The deletion of *fur* did not increase siderophore production. The fact that the depletion of Fur does not significantly impact siderophore production (Fig. 5B), nor increase P*ryhB2* activity under our iron-limited conditions (Fig. 5C), suggests that Fur is completely de-repressed in all our growth conditions, contrary to iron replete condition (Fig. S5C). In contrast, the deletion of *rpoS* led to a marked increase in the concentration of endogenous siderophores in the growth medium, particularly at low pH, where the increase in ENT levels was significantly more pronounced than in the WT strain (Fig. 5B). At neutral pH, the difference was also evident, but the effect was far greater under acidic conditions (1.5-times more ENT at pH 7.5 versus 60-times more at pH 5.5). Additionally, the Δ*rpoS* mutant showed an enhanced iron deficiency response, as indicated by the increased expression of the small RNA *ryhB2* in all three pH conditions tested (Fig. 5C and Fig. S5C). This upregulation was also observed for genes related to both endogenous and exogenous siderophore import, such as *entCEBA, fepA*, and *foxA* (Fig. 5D–F and Fig. S5D). RpoS is known to modulate nutrient acquisition, typically by repressing the expression of various nutrient uptake systems. While at neutral pH iron-uptake systems are already active, the absence of RpoS has only limited effects on siderophore production. Contrary, at low pH, the absence of RpoS results in a more significant effect, where ENT production is strongly increased. This enhanced production of ENT at low pH underscores the stronger iron acquisition capacity in the Δ*rpoS* mutant under these conditions. By repressing endogenous siderophore production, RpoS seems to have a significant role in regulating iron acquisition under acidic conditions, whether directly or through intermediary pathways, leading to a drastic effect on growth under iron starvation.

## Discussion

For successful growth, organisms must adapt to their environments. Given the crucial role of iron for growth, the transcriptional regulation of iron acquisition systems in bacteria must be finely tuned. This is especially critical for pathogens that need to establish infection in environments that are already colonized. This study explored how environmental changes, particularly a decrease in pH, influence the utilization of siderophores by *Salmonella*.

Our competition assays revealed that under acidic conditions, *Salmonella* benefits more from exploiting exogenous hydroxamate-type siderophores than it does at neutral pH. This increased reliance on exogenous siderophores is linked to a stark reduction in the production of endogenous siderophores, monitored in various *Salmonella* strains (Fig. S6), highlighting a drastic adaptation of siderophore production to varying pH changes. This phenomenon has been documented in previous studies for other microorganisms. For instance, Valdebenito et *al*., demonstrated that in *Escherichia* coli, the production of ENT decreases under acidic conditions (Valdebenito et al. 2006). However, this strain, in contrast to our *Salmonella* strain, is also capable of producing other siderophores, such as aerobactin and yersiniabactin. Aerobactin, a mixed hydroxy acid and hydroxamate-type siderophore, reaches peak production at acidic pH, while yersiniabactin, a phenolate-type siderophore, is maximally produced at alkaline pH 8.5. This indicates that siderophore production in *E. coli* is highly influenced by environmental pH and varies with the type of siderophore (Valdebenito et al. 2006).

In addition to affecting siderophore production, environmental pH can also drive bacterial adaptation at the level of siderophore transport. Li et *al*., found that *Acinetobacter baumannii* adapts to pH changes through mutations in the siderophore receptor gene *bauA* (Li et al. 2023). This adaptation allows *A. baumannii* to optimize iron uptake and maintain iron levels under varying pH conditions. To explore whether a similar pH-dependent adaptation occurs in *Salmonella*, we investigated how pH affects the affinity of DFOE-Fe for its transporter FoxA. However, our results revealed no significant change in DFOE-Fe binding to FoxA across different pH levels (Fig. S4).

In addition, pH can also influence the affinity of iron for the siderophore by modifying their chelating properties. ENT, a catecholate-type siderophore, is known to have the highest iron affinity at neutral pH (pFe at pH 7.5 = 35) among all the siderophores described (Harris et al. 1979). However, Valdebenito et *al*. found that this affinity decreases under acidic conditions (pFe at pH 5.5 = 25). In contrast, hydroxamate siderophores like DFO maintain relatively stable iron affinities across different pH levels (pFe at pH 7.5 = 27 and pFe pH at 5.5 = 22) (Valdebenito et al. 2006). Our competition assays, where ENT and DFOE were added simultaneously at equal concentration, revealed that *Salmonella* favors ENT over DFOE even under acidic conditions. The superior iron-binding capacity of ENT makes it the preferred siderophore when both are available, underlining yet again that the stark dependency on exogenous siderophore at acidic pH is solely due to reduced ENT/SLC production. However, we were unable to directly compare the WT and the Δ*entC* mutant strains within the same experiment because the ENT produced by the WT would have been utilized by the mutant, potentially obscuring the true experimental outcome. A similar issue arises when comparing the WT strain to the Δ*rpoS* mutant, which overproduces ENT and is expected to outperform the WT. In this case as well, the excess ENT from the Δ*rpoS* mutant could enhance the performance of the WT strain, thereby confounding the results.

Upon exposure to low pH, *Salmonella* activates the alternative sigma factor RpoS, which orchestrates the acid tolerance response by upregulating the expression of numerous acid shock proteins but also altering nutrient acquisition, thereby enabling the pathogen to survive in acidic environments (Ihnat et al. 2002). Our data suggest that in iron-depleted and acidic environments, RpoS but not Fur reduces siderophore production and thereby increases *Salmonella’s* reliance to exogenous siderophores. The exploitation of exogenous siderophores may allow *Salmonella* to avoid the energy costs associated with producing its own siderophores. This emphasis on energy conservation likely explains why the deletion of *rpoS* positively impacts endogenous siderophore production. The loss of RpoS has already been shown to enhance *Salmonella*’s ability to scavenge for limited nutrients (Notley-McRobb et al. 2002). While this strategy may be less critical at neutral pH, it may become especially important under acidic conditions, where conserving energy is vital for enduring the environmental stress and represent an evolutive response to stress conditions. While our data suggest the implication of RpoS in the down-regulation of ENT at low pH, deletion of *rpoS* may lead to highly pleiotropic phenotype. Further investigations are needed to elucidate the exact regulatory mechanism by which RpoS interacts directly or indirectly with the promoter region of siderophore production and uptake.

Furthermore, while our expression data suggest no difference in ferrous iron uptake system expression at low pH, this does not rule out increased uptake due to the higher solubility and thus availability of Fe²⁺ in acidic conditions. Even so, enhanced ferrous iron acquisition alone did not allow *Salmonella* to outcompete the strong advantage conferred by high-affinity siderophore-iron complexes under iron-limited conditions. Thus, efficient ferrous iron uptake remains insufficient for competitive growth when siderophores are present and actively sequestering iron.

While this study focuses on pH, the gastrointestinal tract is a highly complex environment where many variables—such as oxygen levels, microbial community composition and host factors—can influence *Salmonella’s* iron uptake strategies. We have highlighted pH as an important variable, but oxygen also has a significant effect on iron chemistry and siderophore utilization, and remains an important area for future research. Importantly, the central role of siderophores in iron acquisition in the anaerobic parts of the gastrointestinal tract is supported by their increasingly documented usage in strict anaerobes, reinforcing that these systems are crucial for survival across diverse gut conditions (Rocha and Krykunivsky 2017, Zhu et al. 2020, Spiga et al. 2023, Rodríguez-Daza et al. 2025).

Understanding the conditions under which *Salmonella* utilizes endogenous or exogenous siderophores can provide critical insights for designing targeted therapies. Despite the challenges in pinpointing specific targets, knowing how *Salmonella* adapts its iron acquisition strategies offers valuable information for developing effective treatments. Future research should focus on exploring other environmental clues that could affect iron acquisition mechanisms and thereby offering a better understanding versatility of *Salmonella’s* iron uptake strategies and thereby informing future therapeutic approaches.

## Materials and methods

### Bacterial strains and growth conditions

A full list of all bacterial strains used in this study is provided in sup. Table S1. *Salmonella* strains used were based on sME51, a prototrophic *hisG*^Leu69^ derivative of *Salmonella enterica* serovar Typhimurium SL1344 (Claudi et al. 2014). All strains were cultivated in Lysogeny Broth (LB) (10 g/L tryptone, 5 g/L yeast extract, 5 g/L NaCl) or Minimal Medium (MM) (15 mM NH_4_Cl, 1.5 mM K_2_SO_4_, 3 mM KH_2_PO_4_, 1 mM MgCl_2_, 0.4% glycerol). For pH adjustments, 100 mM HEPES was used to achieve pH 7.5, while 100 mM MES was used for pH 6.5 and 5.5 and filter-sterilized. Media were supplemented with 90 µg/ml streptomycin, and 50 µg/ml kanamycin was added when a plasmid reporter was present. *Escherichia coli* JKe201 was grown in LB supplemented with 100 µM diaminopimelic acid (DAP), and 50 µg/ml kanamycin was added when required. Cultures were incubated at 37°C with shaking at 220 rpm.

### Plasmids and genetic engineering of bacterial strains

All primers and plasmids used in this study are listed in sup. Tables S2 and S3. All plasmids used in this study are low-copy plasmids and carried a kanamycin resistance cassette to ensure their maintenance in bacterial cells. Gene reporter plasmids contained candidate gene promoters fused to a *gfp-ova* reporter to assess specific gene expression in response to experimental conditions as described here (Cunrath and Bumann 2019). For strain differentiation during competition assays, low-copy episomal pSC101-derivatives were employed. These plasmids enabled the constitutive expression of either dTomato or YPet from the P*_ybaJ_* promoter, which facilitated tracking the proportions of different strains (Claudi et al. 2014, Cunrath and Bumann 2019). Gene deletions were obtain using two consecutive single-crossovers, incorporating positive selection for kanamycin resistance and a double negative selection against SacB levan sucrase-mediated sensitivity to sucrose and the expression of the I-SceI endonuclease (Cianfanelli et al. 2020). The engineered strains preserved a nonapeptide with the first five and last four amino acids, including the stop codon, as described here (Cianfanelli et al. 2020).

### Prevalence of siderophore-related genes and phylogenetic analysis in *Salmonella*

To analyze siderophore transporter genes across *Salmonella* strains, we followed a structured bioinformatics workflow. Initially, we sorted and downloaded genomes from the BV-BRC database (Wattam et al. 2017). Genomes were first assessed for metadata and contamination using the CheckM statistics provided by BV-BRC. Genomes were removed if they had a CheckM completeness ≤90 or a CheckM contamination ≥5. We then plotted the number of coding sequences versus genome length. For strains within a species this should be a linear relationship. We removed any genomes which deviated from this linear relationship by >500 coding sequences (Horesh et al. 2021, Sharp and Foster 2024). We then filtered these genomes to retain only those with a minimum size of 4 million base pairs. To ensure the most representative dataset, we grouped strains by their unique multilocus sequence typing (MLST) type and randomly selected one strain per group using Python’s *random.choice* function. This approach allowed us to capture the diversity of *Salmonella enterica* strains effectively and resulted in a final dataset of 183 strains, selected from an initial set of 1 146 isolates. For gene detection, we used the ABRicate tool (Seemann 2025) (v1.0.1) with a custom database to identify specific siderophore-related genes in the genome sequences. Genes were considered present only if they met the default ABRicate thresholds of at least 80% identity and 80% coverage; any gene below these thresholds was classified as absent. Phylogenetic analysis was conducted using PhyloPhlAn (Asnicar et al. 2020) (v3.1.1). For each Salmonella genome, protein-coding sequences were predicted using Prodigal (Hyatt et al. 2010) (v2.6.2). Up to 400 universally conserved phylogenetic markers were identified through DIAMOND (Buchfink et al. 2015) (v2.1.12) searches against the UniRef90 database (Suzek et al. 2015). Each mark gene was then individually aligned using MAFFT (Katoh and Standley 2013) (v7.520) and poorly aligned regions were removed using trimAl (Capella-Gutiérrez et al. 2009) (v1.4.1) with default parameters. The resulting high-quality alignments were concatenated into a single multiple sequence alignment. A phylogenetic tree was inferred from the concatenated alignment using maximum likelihood. An initial topology was rapidly estimated with FastTree (Price et al. 2010) (v2.1.11) and the final tree was computed with RAxML (Stamatakis 2014) (v8) using the GTR+G substitution model. The resulting tree was visualized and further modified using iTOL (Letunic and Bork 2021) (v7.0) to enhance clarity and presentation.

### Competition assays

The competing strains carried a low-plasmid with constitutive expression of fluorescent proteins (YPet or dTomato). Pre-cultures were grown overnight in MM at pH 7.5 with 10 μM iron, at 37°C with agitation (220 rpm). Post-incubation, 1 mL of each culture was centrifuged at 9000 rpm for 2 minutes, washed twice with 1X phosphate-buffered saline (PBS), and resuspended to an optical density at 600 nm of 0.1 in 1 mL of 1X PBS. Equal volumes (500 µL each) of the two strains were mixed to create a 1:1 bacterial suspension at OD_600nm_ of 0.1 (OD 0.05 per strain). Fourteen-milliliter tubes containing 1.8 mL of MM at pH 7.5, 6.5, or 5.5, with or without exogenous siderophore (all chemicals are reported in sup. Table S4), were inoculated with 200 µL of the bacterial mixture to achieve a final OD_600nm_ of 10^−6^. Cultures were grown for 48 hours at 37°C with agitation (220 rpm). The total number of cells for each strain was determined by plating on LB agar supplemented with 90 µg/mL streptomycin and enumerating colonies under blue light to distinguish the fluorescent proteins. The competitiveness index (CI) was calculated as CI = output/input, where output is the ratio of mutant to wild-type colonies after 48 hours, and input is the ratio at time zero.

### Quantification of GFP-fluorescence intensity

Overnight cultures in MM at pH 7.5 with 10 μM iron were grown at 37°C with shaking (220 rpm). Following centrifugation (9000 rpm, 2 minutes) and washing twice with 1X PBS, cells were resuspended to an OD_600nm_ of 0.1 in 1X PBS. Twenty microliters of this suspension were added to 180 μL of fresh MM (or LB for P*katE*) at various pH levels (7.5, 6.5, 5.5) with or without iron or exogenous siderophore in a 96-well plate (Greiner, U-bottomed microplate). The plate was incubated at 37°C with shaking in a Tecan microplate reader (Infinite M200, Tecan), with OD_600nm_, GFP (excitation/emission: 488 nm/510 nm), and mCherry (excitation/emission: 570 nm/610 nm) fluorescence measured every 10 minutes for 48 hours. Results from three biological replicates were analyzed, with GFP fluorescence normalized to OD_600nm_ and the maximum value of this normalization used for histograms. Statistical significance was determined with p-values <0.05.

### Quantification of intracellular iron

Pre-cultures were grown overnight in MM at pH 7.5 with 10 μM iron, at 37°C with agitation (220 rpm). Post-incubation, 1 mL of each culture was centrifuged at 9000 rpm for 2 minutes, washed twice with 1X phosphate-buffered saline (PBS). 250 mL Erlenmeyer containing 50 mL of MM at pH 7.5 or 5.5 were inoculated to obtain a final OD_600nm_ of 10^−6^. Cultures were grown for 48 hours at 37°C with agitation (220 rpm). The total number of cells for each strain was determined by plating on LB agar supplemented with 90 µg/mL streptomycin. Iron quantification was performed using previously described method (Baumann et al. 2019). Briefly, bacterial pellets were washed twice with 1X PBS and dried at 55 °C for 48 h. Dried bacterial pellets were resuspended with 35 µL of nitric acid (70%) for 48 h at room temperature and brought to final volume of 500 µL using ultra-pure water and left for an additional 48 h. Total iron content was measured by reducing ferric iron to ferrous iron using thioglycolic acid. Each sample was analyzed in triplicate. A 20 µL aliquot of each sample was added to individual wells of a 96-well flat-bottom polystyrene microplate (Greiner). To each well, 40 µL of saturated sodium acetate (5.5 M, Sigma-Aldrich), 80 µL of chilled distilled water, 10 µL of a 1:10 dilution of thioglycolic acid in water (Sigma-Aldrich), and 10 µL of bathophenanthroline solution (5 mg/mL, Sigma-Aldrich) were added. The plate was gently shaken and incubated overnight at 4 °C in the dark. Absorbance was measured at 535 nm using a Tecan Infinite M200 microplate reader, and iron concentrations were calculated based on a standard calibration curve.

### Ligand binding assay

To determine the dissociation constant (*K_d_*) between Ferri-DFOE and its transporter FoxA in *Salmonella*, strains were cultured overnight in MM at pH 7.5 or 5.5, with 10 μM DFOE added to induce FoxA transporter expression. Complexes of ^55^FeCl_3_-DFOE were prepared with a ^55^Fe concentration of 20 μM and a siderophore ratio of 20:1 in HCL 0.1 N. ^55^FeCl_3_ was purchased from Perkin Elmer (Table S4). *Salmonella* cells at an OD_600nm_ of 0.1 were incubated with ^55^FeCl_3_-DFOE complexes in cold-buffer at pH 7.5 (100 mM HEPES) or pH 5.5 (100 mM MES) for 1 hour at 0°C, with transport inhibited by adding Carbonyl cyanide 3-chlorophenylhydrazone (CCCP). Bacterial cells were recovered by filtration using a Brandel filtration system (M-48 Cell Harvester) and Whatman® glass microfiber filters (Grade GF/B, 460×570 mm). Radioactivity associated with the bacteria was measured with a scintillation counter to calculate the *K_d_*. This binding assay was conducted in triplicate.

### Siderophore production

Overnight cultures grown in MM at pH 7.5 with 10 μM iron were pelleted, re-suspended in fresh MM at pH 7.5 or 5.5 to an OD_600nm_ of 0.01, and incubated with shaking at 37°C for 24 hours. The OD_600nm_ was measured to assess cell growth. Supernatants were analyzed by UV-Vis spectroscopy. Preliminary, calibration curves with pure ENT and its degradation product, DHBS, were established, with absorbance peaks at 330 nm for ENT and 310 nm for DHBS at pH 7.5, and at 317 nm for ENT and 310 nm for DHBS at pH 5.5. Molar extinction coefficients were εENT pH 7.5 = 8100 M^−1^.cm^−1^, εENT pH 5.5 = 2290 M^−1^.cm^−1^, εDHBS pH 7.5 = 7000 M^−1^.cm^−1^, and εDHBS pH 5.5 = 2100 M^−1^.cm^−1^. Absorbance monitored from supernatants were normalized to OD_600nm_ of the cultures. Control experiments compared the absorbance of ENT/DHBS in wild-type strains with those deleted for siderophore production to account for interference from other media components.

### Statistics

Statistical tests were performed with GraphPad Prism (version 10.2.3) as indicated in the figure legends. Figure legends indicate the statistical tests used with all biological replicates depicted in the figures.

## Supplementary Material

uqaf041_Supplemental_Files

## Data Availability

All data are available in the main text or supplementary materials and on https://doi.org/10.5281/zenodo.15276328.
