# Correction to: *Salmonella* relies on siderophore exploitation at low pH

**DOI:** 10.1093/femsml/uqag022

**Published:** 2026-06-22

## Abstract

Bacterial upstream ORFs are far more common than previously realized, and when ribosome-binding sites lie close together, they cause ribosomal occlusion that suppresses translation of the downstream main gene.

This is a correction to: Manon Ferry, Connor Sharp, Isabelle J Schalk, Olivier Cunrath, *Salmonella* relies on siderophore exploitation at low pH, *microLife*, Volume 7, 2026, uqaf041, https://doi.org/10.1093/femsml/uqaf041

In the originally published version of the paper the Graphical Abstract image was missing. This has now been supplied to the paper.

