# Supplementary material for: Salmonella relies on siderophore exploitation at low pH
Source: Microlife. 2026 Jan 14;7:uqaf041. doi: 10.1093/femsml/uqaf041 (PMC12816392; doi:10.1093/femsml/uqaf041)
Supplement: uqaf041_Supplemental_Files [file uqaf041_supplemental_files.zip › supp_LV_MF.pdf]

Supplementary figures

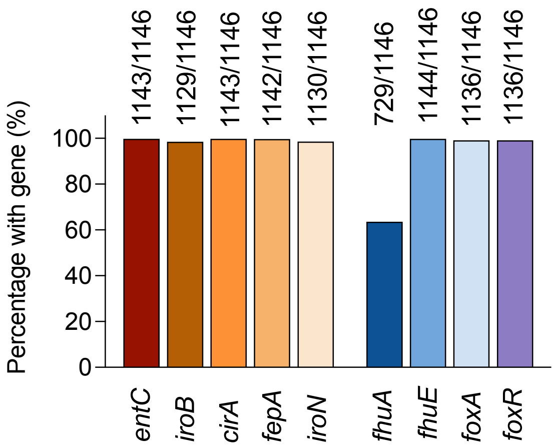

Fig. S1. Conservation of siderophore-related genes among 1,146 *Salmonella* strains from the BV-BRC database. Bars in warm colours indicate the proportion of tested strains carrying genes involved in endogenous siderophore biosynthesis (*entC*, *iroB*) and uptake (*cirA*, *fepA*, *iroN*). Bars in cool colors indicate the proportion of tested strains carrying genes involved in exogenous siderophore uptake (*fhuA*, *fhuE* and *foxA*) and its regulation (*foxR*). These isolates were selected following a structured bioinformatics workflow, which included genome quality assessment, contamination checks, and filtering based on a minimum genome size of 4 million base pairs (see Methods).

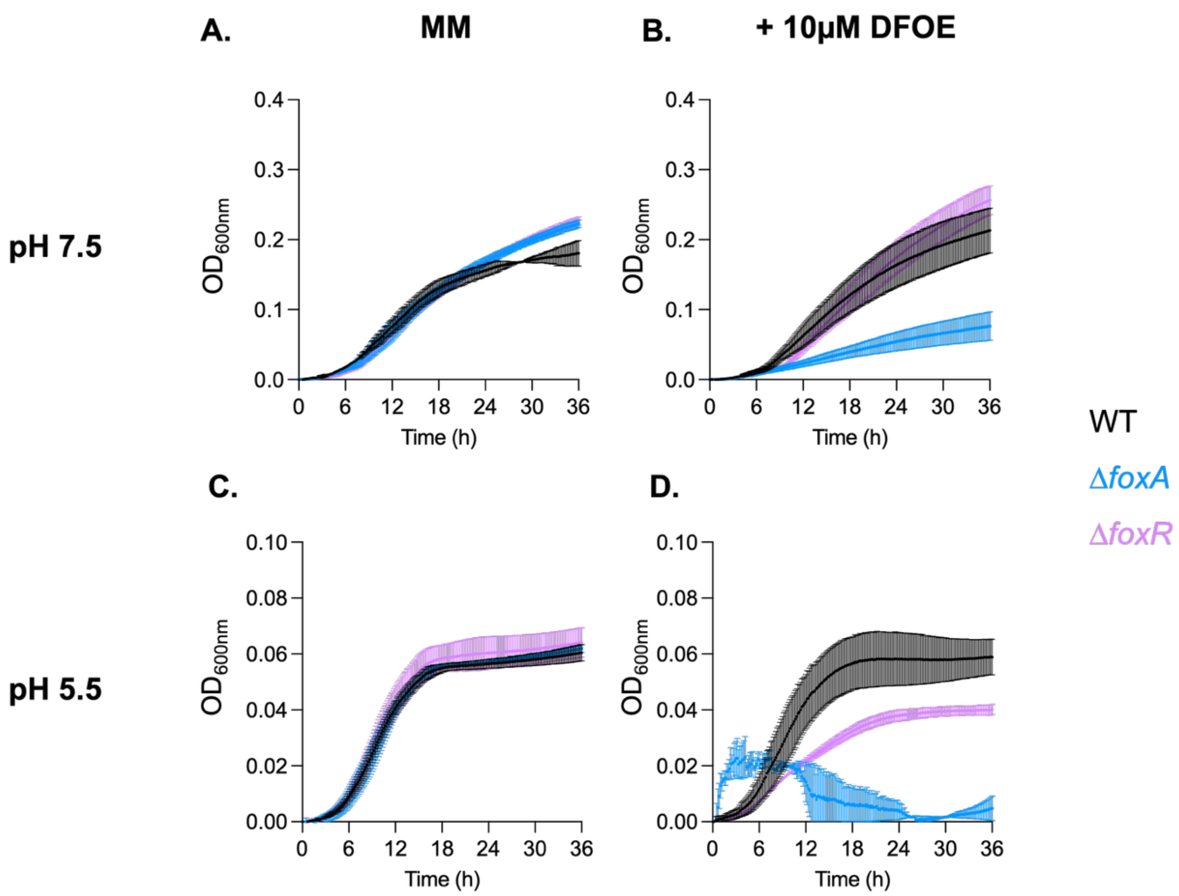

Fig. S2. *Salmonella* WT DFOE-dependent growth kinetics at various pH (7.5 vs 5.5). Bacterial strains were cultivated in iron-depleted medium at either neutral (pH 7.5) or acidic (pH 5.5) conditions, in the absence or presence of 10 µM of DFOE. Growth was initiated at an OD_{600nm} of 0.01 and monitored over time. The WT strain (black) was compared to the Δ*foxA* deletion mutant (blue) which cannot exploit DFOE, and the Δ*foxR* deletion mutant (purple), which is unable to regulate *foxA* expression in response to DFOE. Each growth curve represents the median with the standard error of mean of three biological replicates.

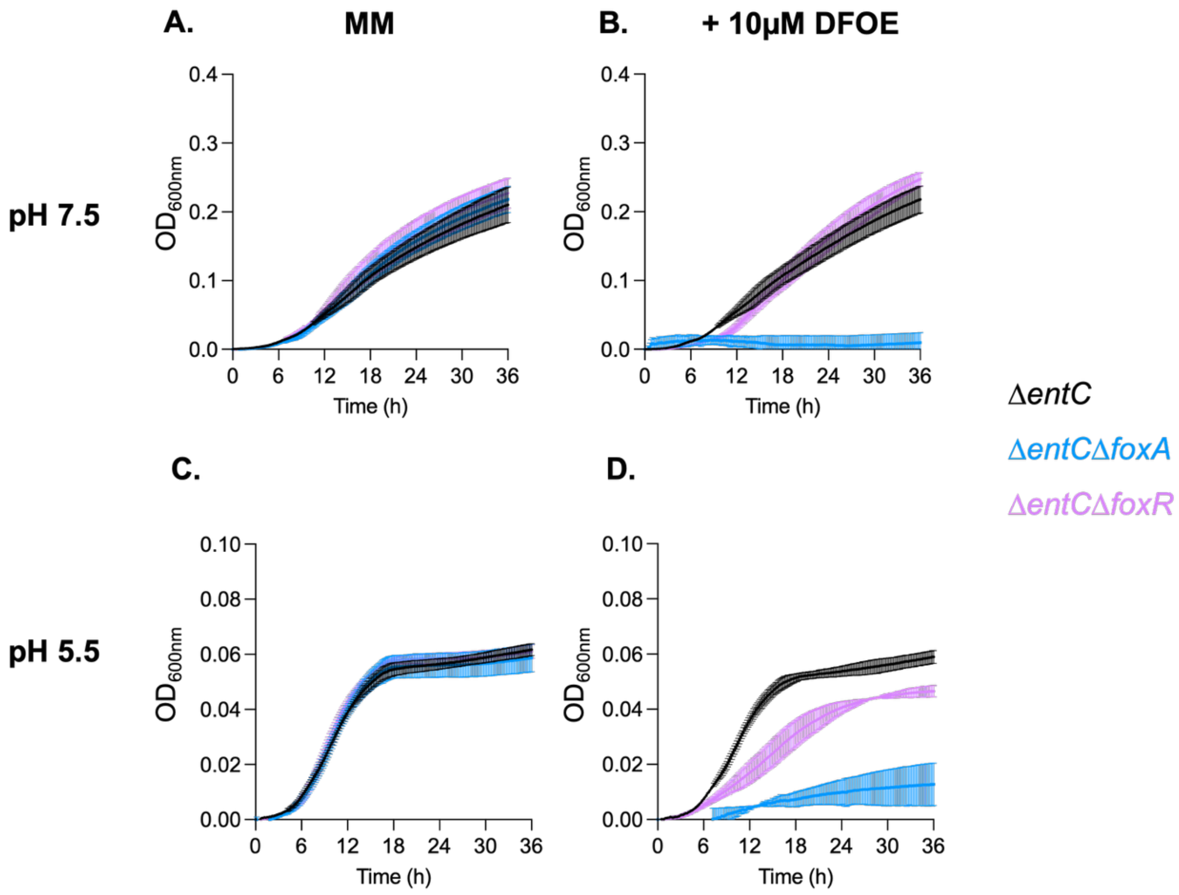

Fig. S3. *Salmonella entC* mutant DFOE-dependent growth kinetics at various pH (7.5 vs 5.5). Bacterial strains were cultivated in iron-depleted medium at either neutral (pH 7.5) or acidic (pH 5.5) conditions, in the absence or presence of 10 µM of DFOE. Growth was initiated at an OD_{600nm} of 0.01 and monitored over time. The Δ*entC* mutant (black), which cannot produce endogenous siderophores but can exploit DFOE, was compared to the Δ*entC*Δ*foxA* double mutant (blue), which lacks both endogenous siderophore production and the ability to exploit DFOE, and the Δ*entC*Δ*foxR* double mutant (purple), which lacks endogenous siderophore production and cannot regulate *foxA* expression in response to DFOE. Each growth curve represents the median with the standard error of mean of three biological replicates.

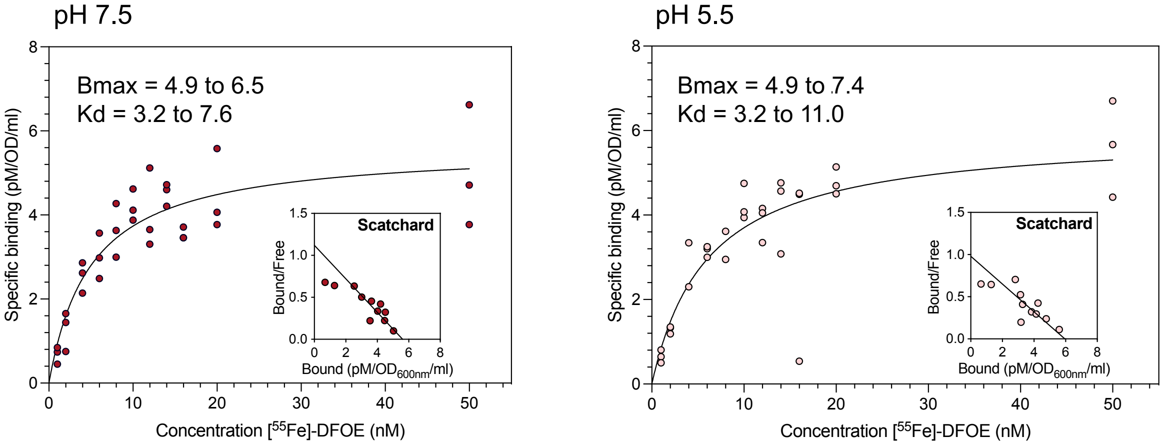

Fig. S4. Binding of Fe-DFOE to FoxA in *Salmonella* in function of the pH (7.5 vs 5.5). Bacterial cells were incubated with a series of concentrations of [^{55}Fe]-DFOE at 0°C for 1 hour in HEPES buffer (pH 7.5) or MES buffer (pH 5.5), with transport inhibited by carbonyl cyanide 3-chlorophenylhydrazone (CCCP). [^{55}Fe]-DFOE binding was assessed by filtration and radioactivity associated with the cells was measured by scintillation counting to determine the dissociation constant (K_d) and the maximum binding capacity (Bmax). The saturation binding curves represent the specific binding of Fe-DFOE to FoxA at both pH conditions. Inset, Scatchard analysis of specific binding. Each data point represents one biological replicate (n = 3).

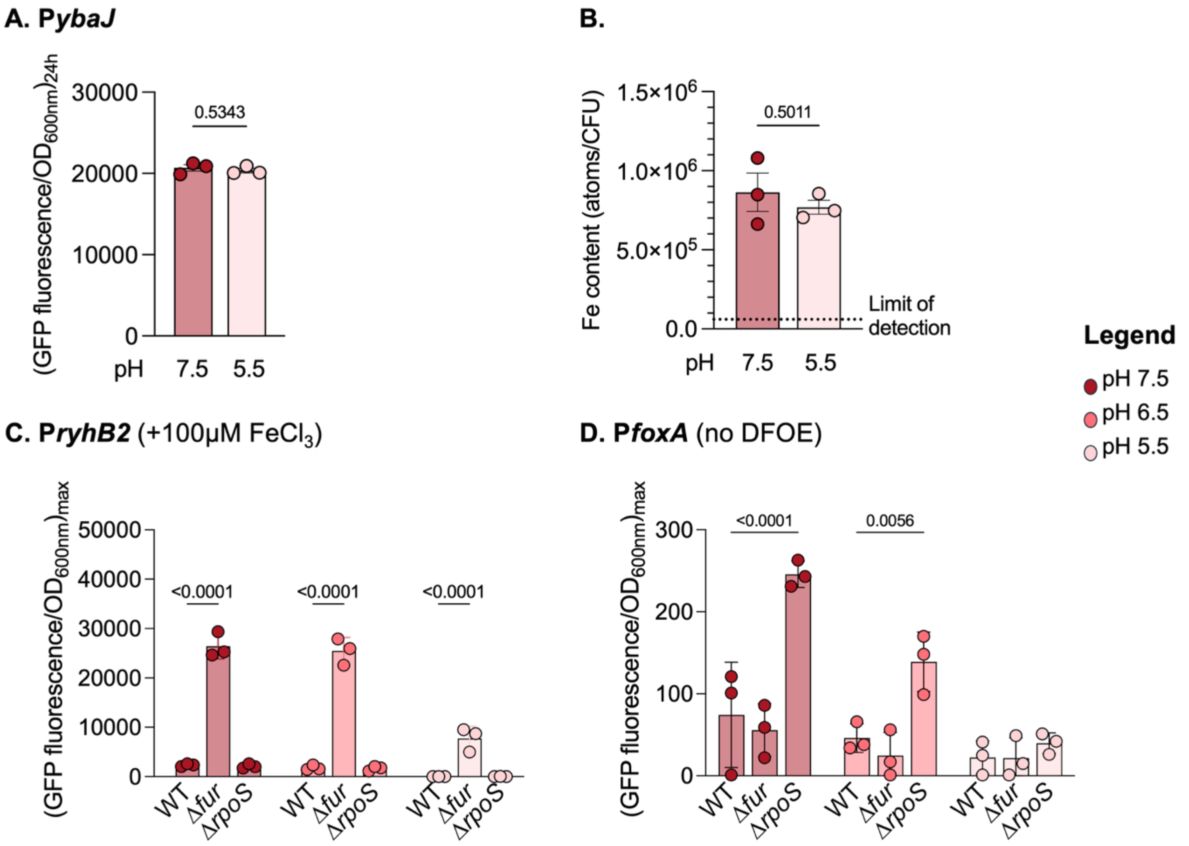

Fig. S5. Transcriptional expression of *ybaJ*, *ryhB2* and *foxA* in *Salmonella* at different pH levels and intracellular iron quantification. A. WT strain carrying PybaJ reporter was cultivated in MM under iron-depleted condition. Fluorescence emission from P*ybaJ-gfp* transcriptional reporters were measured, normalized to OD_{600nm}. The maximum fluorescence signal during growth is shown at pH 7.5 (dark red) and 5.5 (light red). **B.** Strains were cultivated in MM at pH 7.5 (dark red) and pH 5.5 (light red) and intracellular iron concentrations using a colorimetric assay. **C-D.** Strains were cultivated in MM under iron-depleted conditions or supplemented with 100 µM $FeCl_3$ (P*ryhB2*) or 10 µM DFOE (P*foxA*). Cultures were grown at pH 7.5 (dark red), pH 6.5 (medium red), and pH 5.5 (light red). Fluorescence emission from P*ryhB2-gfp* and P*foxA-gfp* transcriptional reporters were measured, normalized to OD_{600nm}. The maximum fluorescence signal during growth is shown. Expression was compared between the WT strain, a Δ*fur* deletion mutant and a Δ*rpoS* deletion mutant. Each point represents one biological replicate (n = 3). Statistical analysis was performed using t-test or a one-way ANOVA followed by Tukey's multiple comparisons test. Exact p-values are reported for all comparisons with $p < 0.05$ considered statistically significant.

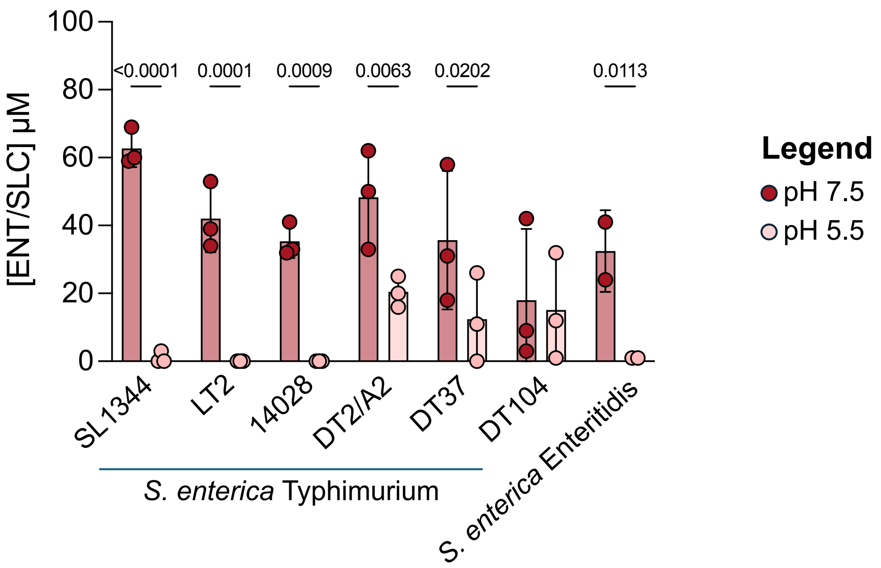

Fig. S6. Endogenous siderophore production (ENT/SLC) by different strains of *S. enterica* in function of pH. Endogenous siderophore concentrations were measured in the supernatant of various *S. enterica* strains after 24 hours of cultivation in MM under iron-depleted at pH 7.5 (dark red) and pH 5.5 (light red). The concentrations of ENT and SLC are shown together as SLC is a glycosylated derivative of ENT. Each point represents one biological replicate (n = 3). Statistical analysis was performed using an unpaired t-test.

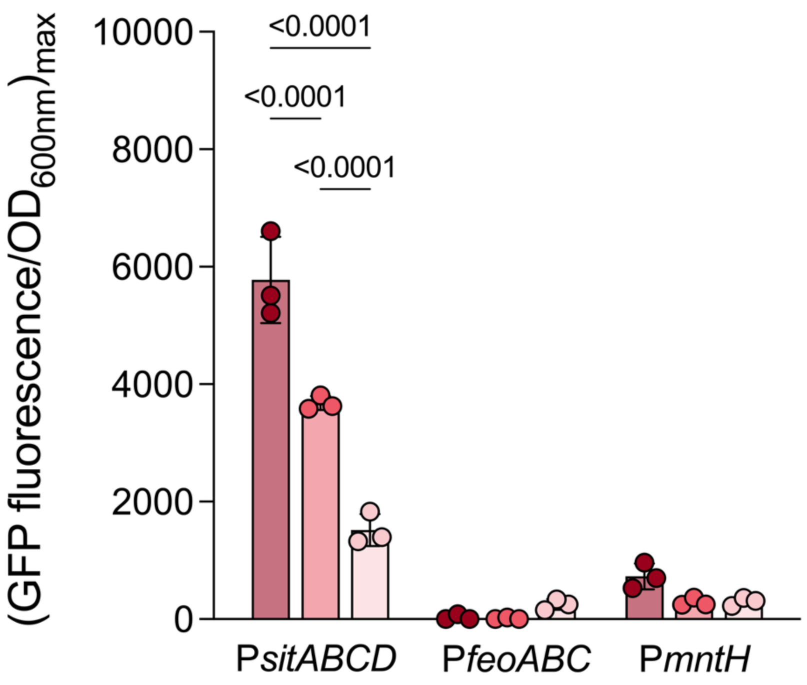

Fig. S7. Transcriptional expression of the ferrous iron uptake systems *sitABCD*, *feoABC* and *mntH* in *Salmonella* at different pH levels. Strains were cultivated in MM under iron-depleted at pH 7.5 (dark red), pH 6.5 (medium red), and pH 5.5 (light red). Fluorescence emission from P*sitABCD-gfp*, P*feoABC-gfp* and P*mntH-gfp* transcriptional reporters was normalized to OD_{600nm}. The maximum fluorescence signal during growth is shown. Each point represents one biological replicate (n = 3). Statistical analysis was performed using one-way ANOVA followed by Tukey's multiple comparisons test. Exact p-values are reported for all comparisons with $p < 0.05$ considered statistically significant.
